# Traumatic Spinal Cord Injury and the Gut Microbiota: Current Insights and Future Challenges

**DOI:** 10.3389/fimmu.2020.00704

**Published:** 2020-05-08

**Authors:** Trisha Jogia, Marc J. Ruitenberg

**Affiliations:** Faculty of Medicine, School of Biomedical Sciences, The University of Queensland, Brisbane, QLD, Australia

**Keywords:** spinal cord injury, neurotrauma, gut dysbiosis, gastrointestinal dysfunction, inflammation, infection

## Abstract

Individuals with traumatic spinal cord injury (SCI) suffer from numerous peripheral complications in addition to the long-term paralysis that results from disrupted neural signaling pathways. Those living with SCI have consistently reported gastrointestinal dysfunction as a significant issue for overall quality of life, but most research has focused bowel management rather than how altered or impaired gut function impacts on the overall health and well-being of the affected individual. The gut-brain axis has now been quite extensively investigated in other neurological conditions but the gastrointestinal compartment, and more specifically the gut microbiota, have only recently garnered attention in the context of SCI because of their vast immunomodulatory capacity and putative links to infection susceptibility. Most studies to date investigating the gut microbiota following SCI have employed 16S rRNA genomic sequencing to identify bacterial taxa that may be pertinent to neurological outcome and common sequalae associated with SCI. This review provides a concise overview of the relevant data that has been generated to date, discussing current understanding of how the microbial content of the gut after SCI appears linked to both functional and immunological outcomes, whilst also emphasizing the highly complex nature of microbiome research and the need for careful evaluation of correlative findings. How the gut microbiota may be involved in the increased infection susceptibility that is often observed in this condition is also discussed, as are the challenges ahead to strategically probe the functional significance of changes in the gut microbiota following SCI in order to take advantage of these therapeutically.

## Introduction

It is well-recognized that traumatic spinal cord injury (SCI) leads to permanent sensorimotor impairments, but perhaps less appreciated is the fact that individuals with SCI also suffer from debilitating multi-system physiological dysfunction (1, 2). For example, autonomic dysreflexia, SCI-induced immune depression syndrome (SCI-IDS) and bladder/bowel dysfunction can all develop after SCI due to a disruption of autonomic pathways between the brain and the spinal cord (3–6). In the context of inflammation and immune function after SCI, the majority of neurotrauma research has focused on defining the activity of specific immune effectors, placing particular emphasis on how these impact on lesion site development. What is much less understood, however, is why the immune response to SCI is aberrant and involves co-existing pro-inflammatory and immunosuppressive elements, both of which are notably not contained to the lesion itself. The overall picture of an obviously multi-faceted and paradoxically-acting immune system therefore remains blurred, including much of its relationship to the secondary sequalae of SCI. Whilst overall mortality has reduced considerably, current treatments do little to combat the more chronic consequences of SCI, including serious visceral comorbidities, and there has been no discernible progress in improving life expectancy and overall quality of life for affected individuals (7–9).

With the increasing recognition of an impaired and/or aberrantly acting immune system, more recent work in the field has shifted its focus toward investigating the possibility of extraneous stimuli or signals that may be influencing the immunological changes that occur after SCI. One logical candidate here may be the gut. The vast microbial communities that reside in the gut (and indeed in other niches in the body) coordinate critical functions for host survival and they have many complex interrelationships with other organs in the body, to the extent that the microbiota is now regarded an organ in its own right. The gut's microbial ecology and intrinsic immune compartment are known to exert considerable influence over basal immunological activity, any perturbations to homeostatic conditions in the gut can therefore have a robust impact on immune function (10–13). How SCI affects this aspect of the gut is only just beginning to be understood. Surveys amongst SCI patients typically reveal gut dysfunction and neurogenic bowel conditions that culminate in reduced intestinal motility, impaired defecation, abdominal pain and associated infection risk as major issues that undermine their overall quality of life, arguably more so than physical paralysis (1, 2, 14, 15). A better fundamental understanding of how the gut contributes to the pathophysiological changes and chronic consequences of SCI is therefore of paramount importance.

## The Gut Microbiota

The pivotal influence and contribution of the gut microbiota to overall health is due in part to the presence of 10^14^ microbes with a taxonomic diversity encompassing bacterial, archaeal and eukaryotic species (10, 12). The microbial inhabitants of the gut are diverse between individuals and while a core microbiota of defined microorganisms does not exist, high-throughput metagenomic sequencing has revealed the reliable presence of 12 bacterial phyla, chiefly *Firmicutes* and *Bacteroidetes*, archaeal phyla, as well as rich fungal communities (10, 16–18). Together, they share a high degree of functional redundancy through a genomic content ~150 times larger than the human genome (19, 20). Diet changes, host behavior and antibiotic treatment throughout life significantly impact on both the gut microbiota and disease susceptibility. At an extreme, work with gnotobiotic mice allude to a compromised ability of the body to effectively manage immunity; this seminal discovery already has wider implications for the use of laboratory animals that have fundamentally misrepresented (or even absent) gut microbiota, in particular when considering the translational value of these experiments (21).

The immune-modulatory capacity of the gut microbiota spans between the production of stimulatory metabolites, and the priming of immune cells that are critical for maintaining the health of the host; however, these influences can become detrimental if the microbial balance is lost. To avoid inappropriate immune activation by “non-self” material, the microbiota is largely kept separate from surveilling host immune cells via physical, biochemical and immunological means (10, 22). There are however certain commensal bacteria that actively interact with immune effectors such as *Bacteroides fragilis*, a member of the *Bacteroidetes* phylum, which directly stimulate regulatory CD4^+^ T cells to enable its own colonization into the epithelium and simultaneously induce beneficial immunosuppression (23, 24). The secretion of immunoglobulin A stimulated by segmented filamentous bacteria (SFB) goes toward limiting the exposure of the epithelium to other pathogenic microbes (10, 25, 26). On the other hand, the pathobiont (i.e., a commensal microbe with the potential to become a pathogenic) *Escherichia coli* similarly adheres to the epithelium, but can trigger the recruitment of Th17 rather than regulatory CD4^+^ T cells, which effectively enhances inflammation in the gut (18, 27). In considering the immunomodulatory potential of metabolites, microbial short-chain fatty acids (SCFAs) derived via anaerobic fermentation have the capacity to exert widespread influence over host cellular function, including epigenetic regulation, stem cell proliferation and gut barrier modulation and, importantly, they also act as potent anti-inflammatory mediators (18, 28). Microbe-directed immune cell manipulation is thought to be necessary for homeostatic immune control, though a push toward pro-inflammatory conditions in disease contexts such as inflammatory bowel disease implicate the pathological potential of these microorganisms if the delicate balance they maintain with the host becomes disturbed.

## Interactions Between the Gut Microbiota and the Central Nervous System

The activity and influence of the gut microbiota is not contained to local immune-gut interactions but extends via critical communication axes to distant organs including the brain. This relationship was made apparent in the association of gastrointestinal disorders with psychiatric conditions, as well as in multiple cases where antibiotic treatment modulated disease outcomes in the central nervous system (CNS) in sterile contexts (18, 29–31). Specific routes of direct communication include afferent fibers from the enteric nervous system (ENS; the intrinsic neural network of the gut), autonomic signaling and humoral pathways such as the hypothalamic-pituitary-adrenal axis and enteroendocrine/mucosal immune system communications (32). Observations that diet-induced changes in the intestinal microbiota were accompanied by increased exploratory behavior in mice suggest that there may be additional pathways for communication with the brain that are independent of the aforementioned routes and instead rely on microbial-derived factors interacting with the CNS (29).

Effects of the microbiota-gut-brain axis have also been implicated in CNS injury and diseases. Investigations of blood-brain-barrier (BBB) development revealed that germ-free mice have increased BBB permeability, which renders the CNS vulnerable; this phenotype was only rescued with the introduction of normal microflora (33). In autism spectrum disorders, a reduced integrity of the BBB, in addition to abnormal neural development and altered gene expression, has also been linked to the gut microbiota (34). The use of germ-free mice has increased appreciation of how the microbiota shapes neuroinflammation, including in the context of experimental autoimmune encephalomyelitis (EAE—the animal model of multiple sclerosis), with both pro- and anti-inflammatory effects (29, 35). Here, germ-free and antibiotic-treated mice show reduced EAE severity compared to normal mice (36), whilst the introduction of segmented filamentous bacteria (SFB) into the gut of these germ-free mice was sufficient to instigate EAE, reinstating the ability of these mice induce Th17 cells (35, 37). Colonization of the gut with *B. fragilis* provided greater protection, however, from EAE symptoms through increased regulatory T cell activity (36, 38). The critical involvement of the microbiota in CNS disease appears recapitulated in humans, as alterations in the microbiome of multiple sclerosis patients correlated with specific gene expression patterns that direct host immune activity (39).

In the context of traumatic CNS injuries, pivotal work by Houlden et al. (30) showed that the gut microbiota undergoes significant change (i.e., gut dysbiosis) after experimental stroke and traumatic brain injury (TBI). A disturbance of the microbiota-gut-brain axis is thought to underpin the symptoms of abdominal pain, intestinal immobility and gastric ulcer formation that occur in these patients following injury (31). After an insult to the brain, a significant loss of cholinergic neurons in the gut submucosa, accompanied by an upregulation of noradrenaline from sympathetic terminals in the gastrointestinal tract, dysregulate the gut microbial content (30, 40). Changes in the gut microbiota that favor pathogenic bacteria (gram-negative species of *Bacteroidetes* and *Proteobacteria*) over beneficial species (from the *Firmicutes* phylum) can be observed as early as 2 h post-injury and persist for a week; interestingly, these alterations in microbial abundance are predictive of the lesion volume and associated behavior deficits in an almost dose-dependent manner (41). To counter gut dysbiosis after TBI, antibiotic treatment targeting pathobionts as well as probiotic interventions that support anti-inflammatory activity have been successful in decreasing pathology in the gut, thereby conferring neuroprotection. Whilst these findings emphasize the role of the microbiota-gut-brain axis in CNS injury, the exact mechanisms behind these observations and the associated clinical implications have not been addressed.

## Current Research into SCI and the Gut Microbiota

The gut microbiota has also been rapidly gaining interest for potential “disease-modifying” effects in SCI (42). Whilst this is unsurprising given the obvious parallels between TBI and SCI, it is important to consider the direct innervation of the gut from the spinal cord, and how this may be differentially affected between these conditions as well as within SCI itself based on the neurological level of the lesion. Sympathetic nerve fibers providing autonomic input into the ENS originate from the thoracic region of the spinal cord, whilst visceral sensory afferents carrying feedback from the gut synapse with spinal cord neurons that eventually transit to the brain (43). Afferent vagal fibers also report to the brain, specifically informing it of the conditions of the intestinal environment (18). Interruption or loss of control over these various pathways and feedback loops push the intrinsic ENS circuits away from homeostasis, and this autonomic imbalance in part explains why SCI patients also suffer from severe gut immobility, fecal retention and increased risk of infections, all of which culminate in a considerably reduced quality of life (1). The impact of SCI on the gut microbiota and the subsequent consequences on inflammation and immune function are only now beginning to be systematically interrogated (see Table 1 for a summary overview).

**Table 1:** A summary of gut microbial changes after SCI in pre-clinical and human investigations.

	**Study details**	**PCR gene primers**	**Microbial changes (vs. control) Phyla + lower taxonomic ranks**	**Intervention/Treatment**	**References**
**Pre-clinical studies**	**Animal**: Female C57BL/6 mice **SCI:** 75 kdyn T9 contusion (cont.) SCI **Controls:** T9 Laminectomy + naïve **Timepoints:** ≤ 28 days hard enterSeparately housed, no antibiotics. Food intake equilibrated across all animals.	16s rRNA V4–V5 515F806R	↑*Firmicutes* ↑ (o) *Clostridiales* ↓ *Bacteroidetes* ↓ (o) *Bacteroidales*	**VSL #3 probiotic** ↓ Gut dysbiosis ↑ Functionalrecovery	(54)
	**Animal**: Adult female Fischer rats **SCI:** moderate-severe T9 cont. SCI (weight drop: 10 g rod from 25.0 mm) **Controls:** T9 Laminectomy **Timepoints:** 8 weeks (wks) Co-housed in injured + non-injured pairs, 7-day gentamicin treatment. *Ad libitum* access to food and water.	16s rRNA V4 Unknown primers	= α diversity *Actinobacteria* ↑ (f) *Bifidobacteriaceae* ↑ (s) *B. choerinum Firmicutes* ↑ (f) *Clostridiaceae* ↑ (s) *C. disporcum* ↓ (s) *C. saccharogumia* (f) *Lactobacillaceae* ↑ (s) *L. intestinalis*	–	(50)
	**Animal**: Adult female C57BL/6 mice **SCI:** 50 kdyn T9 cont. SCI **Controls:** T9 Laminectomy **Timepoints:** ≤ 6 weeks Co-housed in exp. group, no antibiotics	16s rRNA V3–V5 (V4) Unknown primers	↑ Increased bacterial load ↓*Firmicutes* ↑*Bacteroidetes* ↑*Proteobacteria*	**PDE4B**^**−/−**^ **KO mice** ↓ Gut dysbiosis ↓ Neuroinflammation ↑ Functionalrecovery	(53)
	**Animal**: Adult female C57BL/6 mice **SCI:** 70 kdyn T10 cont. SCI **Controls:** T10 Laminectomy **Timepoints:** 28 days No antibiotics. *Ad libitum* access to food and water.	16s rRNA V3–V4 338F 806R	↑α diversity *Firmicutes* ↓ (o) *Lactobacillales* ↓ (g) *Lactobacillus* ↑ (o) *Clostridiales* ↑ (f) *Lachnospiraceae Actinobacteria* ↓ (o) *Bifidobacterialis*	**Melatonin** ↓ Gut dysbiosis ↓ Leaky gut ↑ Functionalrecovery	(55)
	**Animal**: Adult female Lewis rats **SCI:** 125 kdyn unilateral C5 cont. SCI **Controls:** C5 Laminectomy and naïve **Timepoints:** preinjury, 3 days, 4 weeks Co-housed in exp. group; no antibiotics. *Ad libitum* access to food and water.	16s rRNA V4 Unknown primers	↑ α diversity in all groups at 3 dpi Significantly different OTUs (g/s level): 155 = SCI vs. healthy 40 = SCI vs. sham *Analysis of phylogeneticdifferences in supplementary data*	**Fecal Transplant** ↓ Gut dysbiosis ↓ Anxiety-likebehavior	(45)
**Human studies**	**SCI:** AIS grade A Cont. SCI **Control:** Healthy individuals **Further comparisons:** Upper motor neuron (UMN) + lower motor neuron (LMN) bowel syndrome **Timepoints:** ≥1 year post-injury 1–3 weeks standard diet, 3 weeks no antibiotics	16s rRNA V4 515F 806R	*Firmicutes*↓ (g) *Pseudobutyrivibrio* ↓ (g) *Dialister* (UMN) ↓ (g) *Megamonas* ↓ (g) *Marvinbryantia* (UMN vs. LMN) ↓ (g) *Roseburia* (LMN)	–	(44)
	**SCI:** AIS grade A SCI **Control:** Healthy males **Further comparisons:** Quadriplegia (quad) vs. paraplegia (para) **Timepoints:** ≥ 6 months post-injury 2 weeks standard diet, 1 month no antibiotics	16s rRNA V3–V4 338F 806R	↓α diversity ↓*Firmicutes* (Quad vs. Healthyand Para) ↓ (g) *Dialister* ↓ (g) *Megamonas* ↓ (g) *Eubacterium* ↓ (g) *Subdoligranium* ↓ (g) *Faecalibacteria* (Quad) ↑ (g) *Blautia* ↑ (g) *Lachnoclostridium* ↑ (g) *Phascolarctobacterium* (Para) *Bacteroidetes* ↓ (g) *Prevotella* ↑ (g) *Bacteroides* ↑ (g) *Parabacteroides* (Para) ↑ *Proteobacteria* ↑ (g) *Escheria/Shigella* ↑*Verrucomicrobia*	–	(14, 52)* *Same quadriplegic patient cohort

The first report of changes in the gut microbiome of SCI patients identified a specific reduction of beneficial butyrate-producing microbes of the *Firmicutes* phylum at 12 months or more post-injury compared to healthy controls (44). Although this work was primarily descriptive via the use of 16S ribosomal RNA (rRNA) genomic sequencing, it was suggested that this microbiome profile may be pointing toward a reduced immunomodulatory metabolite content of the gut. Later pre-clinical work in a thoracic level 9 (T9) contusion SCI mouse model by Kigerl et al. (54) reported that SCI increases gut permeability 1 week after injury, and it was postulated that, similar to stroke (40), this may allow for bacterial translocation to distant organs (45). This landmark study also sequenced 16S rRNA, which was extracted from fecal samples of mice with a moderate-severe T9 contusive SCI up to 28 days post-injury. Their results showed that the bacterial orders *Bacteroidales* decreased while *Clostridiales* significantly increased over time post-SCI. Although there is no doubt that profound changes to the gut microbiota did occur in these SCI mice, a consideration around this study is that the experimental design did not include fecal samples from sham-operated controls beyond the sub-acute phase (>7 days post-SCI). The 16S rRNA sequencing results for these mice were further presented as pooled data rather than being split between the acute (0–3 days) and sub-acute (5–7 days) phases post-surgery. A more recent study by Schmidt et al. (45) showed acute effects of surgery (i.e., laminectomy) and/or anesthesia on the gut microbiome, albeit in rats, and others have reported rapid and profound shifts in the gut microbiome profile of poly-traumatized human patients with no documented history of neurological injury (46). Going forward, the impact of trauma itself, SCI severity, lesion level and possible interspecies differences therefore all require careful investigation as to how they impact on the gut microbiota and, if so, for how long these changes persist or perhaps even diverge with time (46–49). This becomes particularly important when exploring correlations between select changes in the gut microbiota and the neurological outcome.

A separate study by O'Connor et al. also examined differences in microbial content of the gut following T9 contusion SCI in rats (50). They detected significant modifications in the gut microbiome after SCI during the intermediate/chronic phase of SCI (8 weeks post-injury) as compared to the sham-operated control group. Somewhat counterintuitively perhaps is that this study found a greater prevalence of *Lactobacillus intestinalis*, a lactic acid-producing probiotic bacterial species generally considered to be beneficial. By the same token, the microbiota of SCI animals also showed unexpected post-SCI rises in certain *Clostridaiceae* and *Bifidobacterium* species that are primarily thought to be beneficial. It may be that the activity of these commensal bacteria becomes pathogenic (and/or of lesser influence) within an inflammatory environment (51). Certainly, pro-inflammatory cytokines such as IL-1β, IL-12 and MIP-2 were significantly elevated in intestinal tissue 4 weeks after SCI, the extent of which was also correlated with a reduction of beneficial butyrate-producing bacteria in the gut, which falls in line with previous human SCI work (44). It is important to note, however, that all animals in this study received a 7-day course of gentamicin treatment following surgery. How this and also the use of general anesthesia in experimental studies impacts on the gut microbiota, including the shaping of any SCI-associated changes therein, remains unclear. Whilst the study by Kigerl et al. (54) therefore may provide more specific insights how traumatic SCI itself impacts on the gut microbiota, the findings of O'Connor et al. (50) are still of significant translational value given that most human patients undergo surgery and typically receive prophylactic antibiotic as well as probiotic treatment after their injury.

Several more recent reports have attempted to better define the consequences of SCI-induced changes in the gut microbiota, linking these directly to specific bacterial types that could be directly therapeutically targeted. For instance, in a Chinese cohort of male SCI patients, Zhang et al. (14) reported that the overall diversity of the gut microbiota was significantly reduced 6 months after SCI compared to healthy controls. Amongst a spectrum of changes in bacterial phyla and genera and an overall decrease in microbial diversity after SCI, these authors found that *Bacteroides*, a genus of the *Bacteriodales* order, increased with SCI (14); they also observed an increase in the abundance of bacteria from the *Proteobacteria* and *Verrucomicrobia* phylum. These changes were directly compared with aspects of neurogenic bowel dysfunction as well as the extent of physical paralysis, which again revealed more specific microbial alterations. A more recent follow-up investigation by this group correlated these established changes in the microbial profile to the serum lipid profiles of this patient cohort (52). Another investigation in mice by Myers et al. also characterized SCI-induced gut dysbiosis, noting a significant increase of the *Proteobacteria* phylum at 6 weeks after injury compared to an uninjured control group, which is in agreement with human SCI findings and perhaps suggests a bias toward gram-negative endotoxin-containing bacteria as drivers gut pathogenesis in this condition (53). The findings of this study also pointed toward a reduction in *Firmicutes*, along with an increase in *Bacteroidetes* phyla. Genetic ablation of the phosphodiesterase PDE4B prevented these changes in bacterial phyla, which coincided with improved functional recovery via inflammatory modulation. An investigation by Jing et al. (55) measured an overall increase in bacterial diversity in SCI mice [which goes against some human SCI microbiome analysis (14)], in particular a relative increase in the abundance of *Clostridiales*, as was found in previous work (54), and a decrease in *Lactobacillales* and *Bifidobacteriales*. Daily melatonin treatment post-injury appeared to reverse some of these changes, and this was correlated with a more favorable cytokine profile and improvements in gut barrier integrity and functional recovery (55). Lastly, the study by Schmidt et al. that was alluded to earlier documented transient changes in the gut microbiota of rats with a unilateral mild cervical contusion SCI, which occurred as early as 3 dpi before resolving by 4 weeks, and correlated these changes with anxiety-like behaviors (45). Treatment of these rats with fecal transplants from naïve animals resulted in a normalization of the gut microbiota based on 16S rRNA sequencing results, and also prevented the onset or development of anxiety-like behaviors. No improvements in lesion pathology and locomotor recovery were observed in association with this intervention (45). Interestingly, the study by Kigerl et al. (54) showed that the extent of neuroinflammation at the site of SCI could, at least partly, be ameliorated with the therapeutic use of the probiotic VSL #3, which contains lactic acid-producing bacteria from the *Lactobacillus* and *Bifidobacterium* genera. Whilst the root causes that drive SCI-induced gut dysbiosis remain unknown, and also how VSL #3 provides neuroprotection at the lesion site, this finding clearly holds promise for clinical translation. It also emphasizes the point that either preventing gut dysbiosis or, alternatively, restoring the composition of the gut microbiota to a pre-injury state may not necessarily lead to beneficial outcomes, but rather that introduction and/or boosting of beneficial microbial communities may be required to skew the inflammatory response toward one that improves the neurological outcome.

Integrating all these specific microbial alterations at various taxonomic ranks and the functional significances of these will now form the next challenge, especially given the vast amount of data that is typically acquired from sequencing studies. On the whole, most investigations have reported a decrease of bacterial taxa in the *Firmicutes* phylum that occasionally coincides with an increase of the *Bacteroidetes* phylum. In combination, this may be indicative of a SCI-associated adjustment in the “*Firmicutes*:*Bacteroidetes*-ratio.” It should be noted that the studies of Kigerl et al. (54) and O'Connor et al. (50) seemingly reported opposing results here, but these are likely attributable to experimental deviations. Specifically, Kigerl et al. (54) housed their mice individually to avoid coprophagia whilst animals were co-housed in the study by O'Connor et al. (50). As mentioned earlier, O'Connor et al. (50) also prophylactically gave their SCI rats 7 days of gentamicin treatment whereas the study by Kigerl et al. (54) avoided the use of antibiotics altogether. More broadly speaking, recent pivotal work by The Human Microbiome Project showed that the *Firmicutes*:*Bacteroidetes*-ratio may be more reflective of an organism's “microbial equilibrium” and therefore not necessarily as suitable a measure of dysbiosis between individuals as previously thought (56). The *Proteobacteria* phylum also appears to increase in abundance in both a pre-clinical and SCI patient setting. Given that certain *Proteobacteria* genera have been implicated in driving peripheral inflammation (57), future studies should therefore derive a clearer putative mechanism for this phylum in the context of SCI-associated pathology.

Taken together, the above-listed exploratory studies have been instrumental in substantiating the association between the gut microbiome and SCI-associated pathology, although the findings remain correlative for the most part and the drivers of dysbiosis are still currently unknown. All of these investigations employed 16S rRNA gene sequencing to map out bacterial diversity of the gut via the generation of big genomic datasets. It is important to recognize that, when used in isolation, this technique has some major caveats: (1) archaeal and fungal communities are omitted from these analyses and, perhaps more importantly, (2) the identification of specific organisms may not necessarily be conducive to defining the causes of dysbiosis, altered gut function and its wider peripheral consequences. This may run the risk of potentially convoluting our understanding of how certain microorganisms drive and/or link to pathophysiological changes. The reliance that this type of analysis places on designating operational taxonomic units of interest overlooks the global metabolic/physiological potential of the gut microbiota as a whole, which ultimately may provide a more informative and complete perspective on gastrointestinal activity after SCI. It should also be noted that examining both the murine and human microbiomes at a genus/species level may not be appropriate at times, and interrogating the broader functional perspective of the microbiota instead, perhaps via the use of enterotypes, may be a more applicable and translatable approach in this field (58).

## Enterotypes as a Way Forward to Interpret the Significance of Changes in Microbiota Between Conditions and Species?

A multitude of techniques, experimental design options and analysis strategies have been recommended to better resolve the functional profile of the gut microbiota in SCI and the putative implications thereof [reviewed in Kigerl et al. (59)]. One additional approach may, however, be to consider the entire microbiota of an organism as a whole via the use of global classifications, also known as enterotypes (58). This stratification strategy aids in removing bias that researchers often place on changes in specific microbial genera/species, which may be overstating the functional relevance of these (60, 61). The human gut microbiome was the first to be stratified into three enterotypes based on bacterial compositional clustering around a central/driver taxon, with profiles aligned around particular functional characteristics, such as the synthesis of different vitamins and various metabolic activities (60). Enterotypes have been claimed to represent the majority of inter-individual diversity in humans as opposed to a continuum of microbial differences and, notably, certain abundant physiological functions were associated with relatively rarer bacteria genera (60). The delineation of specific enterotypes continues to receive much scrutiny, however, with some recent work proposing a gradient of microbial variation, and others being concerned by the inherent risk of oversimplification with a stratification model (61–63). It is nonetheless evident that analyzing global microbial patterns is likely to prove quite informative given the overlap in enterotypes observed in humans and model organisms (58, 64). Hildebrand et al. (64) indeed revealed the presence of such enterotypes in various strains of laboratory mice, with a low-richness cluster that was dominated by *Bacteriodetes* (similar to the human equivalent “enterotype 1”) and a high-richness cluster was populated with *Ruminococcaceae* (similar to the human equivalent “enterotype 3”). The microbial richness of these murine clusters was also found to be associated with varying levels of the calprotectin protein, a marker of intestinal inflammation, suggesting that certain mice may more readily induce inflammation depending on their gut enterotype (64). The presence of similar enterotypes in humans and laboratory mouse strains therefore demands a greater awareness to be given to enterotypes during experimental design in pre-clinical research, particularly from a translational perspective, as they may not necessarily be reflected in genera/species-specific compositional differences (58). To date, trauma-related perturbations of enterotypes have not been studied in humans or mice. Future work may therefore benefit from profiling potential enterotype-like clustering in animals prior to and after injury, in order to examine the possible impact of identified gut patterns or changes therein, and to also ascertain the impact of an “injury-state” enterotype on whole organism physiology (61).

## The Role of the Gut Microbiota in Infection Susceptibility

SCI patients are highly susceptible to life-threatening infections, and this is often attributed to the peripheral immune depression that many patients experience following their injury, a phenomenon known as SCI-IDS. SCI-IDS manifests as a reduction of circulating leukocytes, acute lymphoid organ atrophy and, in animal models, an increase in bacterial colony-forming units (CFUs) that can be cultured from e.g., the lungs (65, 66). Seminal work by Prüss et al. (65) showed that an adrenal gland removal/transplantation paradigm (which restores only cortical function of the adrenal glands), could achieve a “homeostatic” re-balancing of noradrenaline and glucocorticoid levels. This was found to alleviate the consequences of SCI-IDS after high-level (T1) SCI, with reductions noted in the number of CFUs that could be cultured from the lungs. This study did not establish, however, the critical mechanisms that allow for this “spontaneous pneumonia” to develop, leaving the question of how an imbalance in the aforementioned stress factors leads to an increased presence and/or growth of microbes in the lungs as of yet unanswered. Kigerl et al. (54) suggested that the gut may perhaps “leak” infectious microbes after SCI, and that this may lead abnormal bacterial presence in extraneous tissues such as the lungs; however, the capacity of the gut microbiota to instigate infection through translocation is yet to be proven.

Support for a role of the gut microbiota as a source of disseminating bacteria comes from prior investigations into infection susceptibility following ischemic stroke (40). Here, Stanley et al. (40) demonstrated that airway infections after stroke are only observed in specific-pathogen-free mice (i.e., mice with microbiota but devoid of known pathogens), and not germ-free mice (i.e., mice with no microbiota). Sequencing of the lung microbiome after injury and bacterial tracking experiments led to the conclusion that the microbial source of infection was derived from the gut content. These findings are consistent with observations in other conditions where a translocation of bacteria to the lungs has been described, including sepsis and acute respiratory distress syndrome (67). In their exploration of potential mechanisms driving gut dysregulation after stroke, Stanley et al. (40) showed that the intestinal barrier was “leaky” as a result of increased gut permeability and altered epithelial tight junction distribution. These researchers also suggested that disrupted sympathetic innervation of the gut triggers the movement of commensal bacterial from the gut, which they verified via a reduced post-stroke infection incidence with adrenergic receptor inhibition (40). Taken together, this investigation offered a highly novel concept for the occurrence of airway infections after an acquired CNS insult, which may rationalize the theoretically-coupled incidences of SCI-induced gut dysfunction and dysbiosis with the heightened infection susceptibility in this patient population.

It is interesting to note, however, that although Stanley et al. (40) provided evidence in support of the premise that a disrupted neuronal circuitry instigated gut permeability and dysregulation after experimental stroke, blocking adrenergic signaling only resulted in a decreased bacterial load as opposed to a complete elimination of microbes from the lungs, suggesting the putative existence of other/concurrent mechanisms that add to infection susceptibility. Intestinal damage resulting in the “leaky gut” phenotype may additionally be explained by excessive inflammatory processes that already exist in the context of the original disease/injury occurring in the host. For instance, in graft-vs.-host disease, neutrophils are recruited to the intestinal wall and are responsible for tissue damage via the induction of reactive oxidative species. Their destructive activity appeared dependent on the presence of translocating microbes into the peri-intestinal tissue, as neutrophils were not recruited in germ-free mice (68). Thus, displaced microbes may act as a chemotactic stimulus for inflammatory immune cells, whereby their movement could dictate the site of inflammation. When considering SCI, it is well-established that neutrophils do not just accumulate at the lesion site itself (69), but also in peripheral tissues. Here, systemically circulating neutrophils can cause widespread tissue damage in other organs such as the liver, spleen, lung and kidneys, a phenomenon more generally known as Systemic Inflammatory Response Syndrome (SIRS) (70, 71). It has already been noted that patients with severe SIRS also endure gut dysbiosis (72), so it is tempting to speculate that the early mobilization and priming of neutrophils by a CNS insult like SCI may also deleteriously affect the gut. Alternatively, the immunomodulatory activity of commensal gut microbes and/or the changes therein, as described earlier, may also add to altered systemic immune function. These possibilities are not mutually exclusive.

**Figure 1:**
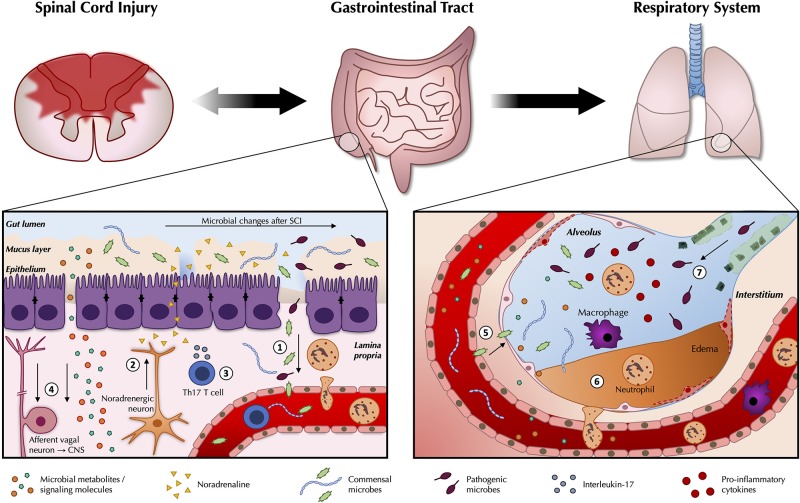
Pathogenic changes in the gut microbiota after traumatic spinal cord injury. As part of the wider systemic response to SCI, inflammatory changes in the gut are likely to contribute to reduced intestinal function and barrier integrity (1). Leakiness of the gut epithelium can dysregulate the microbial community in the gut lumen and allow for bacterial translocation. Release of noradrenaline from post-ganglionic sympathetic terminals is thought to further contribute to gut dysbiosis (2). A greater abundance and/or expansion of specific pathobionts after SCI can induce a Th17 response, which propagates further inflammation (3). Afferent sensory feedback signals report perturbations in the intestinal environment to the brain via the vagus nerve, thereby completing the bidirectional loop between the gut and the central nervous system, while gut microbes release metabolites and signaling molecules such as short-chain fatty acids (SCFAs) that shape peripheral immunomodulatory processes (4). These changes may modulate the immune response to e.g., airway infections. Commensal microbes from the gut may also be able to take up inappropriate residence in the lungs (5). Local inflammatory changes in the lungs in response to SCI may compromise the respiratory epithelium (6), and interfere with local defense mechanisms against extraneous pathogens (7).

Perturbations to normal crosstalk between the gut microbiota and the immune system may disrupt this delicate homeostatic balance and provoke the unwanted residence and/or growth of extraneous pathogens in the airways of the host (11). For example, protection against pneumonia instigated by *S. aureus* is conferred in part by the activity of SFB in the gut which again promote pulmonary Th17 immunity (73). The gut microbiota normally positively regulates host defense against pneumococcal pneumonia by limiting bacterial dissemination, controlling inflammation, and by enhancing the phagocytic function of resident alveolar macrophages; these protective influences of the gut microbiota are dysregulated in germ-free mice (11, 74). These results are also corroborated by experiments in Rag^−/−^ mice (which are deficient in T and B cells), as gut SFB can still instruct innate immune effectors here to resolve infections via the gut-lung axis (75). Lastly, unregulated secretion of anti-inflammatory SCFAs may also negatively interfere with host immunity and play into the pathophysiology of respiratory diseases, as has been documented in a cohort of tuberculosis-suffering patients (76). Studies into the intestinal microbiota of human patients already indicate changes in beneficial butyrate-producing microbes after SCI, warranting further investigations as to how this may play into impaired host immunity (44). Given the high prevalence of airway infections after SCI, a better understanding of how disruption in critical feedback circuits with the gut microbiota can work together with SIRS as a possible propagator of tissue damage may further rationalize the degree of vulnerability patients have toward extraneous sources of infection (i.e., of nosocomial origin) after injury (77, 78) (see Figure 1).

## Conclusion

It is clear that individuals with SCI suffer from severe gastrointestinal dysfunction, the extent of which significantly impacts on their overall quality of life. As the importance of the gut microbiome for overall health and well-being is increasingly recognized, the significance of investigating the impact of SCI thereon is without question. Recent investigations all corroborate evidence that SCI undeniably changes the gut microbiota, and future studies can now aim to more specifically address how altered signaling via the CNS-gut axis may influence outcomes. Moving forward, future studies should aim to engage advanced metagenomic techniques so that the overall immunological and functional influence of the gut microbiota can be evaluated more thoroughly. As gut dysfunction may play a role in the increased infection susceptibility of this patient population, the net influence of changes in the gut microbiota over host immune function after SCI need to be better understood. It will be imperative, however, that all aspects of the gut microbiota are considered here to generate wholistic perspective of immunological dysfunction and microbial alterations after SCI, in order for these to be successfully translated into effective intervention strategies for SCI patients.

## Author Contributions

TJ drafted the manuscript. MR provided critical feedback. Both authors contributed to editing and approved of the final manuscript.

## Conflict of Interest

The authors declare that the research was conducted in the absence of any commercial or financial relationships that could be construed as a potential conflict of interest.
